# Imperatorin Interferes with LPS Binding to the TLR4 Co-Receptor and Activates the Nrf2 Antioxidative Pathway in RAW264.7 Murine Macrophage Cells

**DOI:** 10.3390/antiox10030362

**Published:** 2021-02-27

**Authors:** Mei-Hsuen Huang, Yu-Hsien Lin, Ping-Chiang Lyu, Yi-Chung Liu, Yuan-Shiun Chang, Jing-Gung Chung, Wei-Yong Lin, Wen-Tsong Hsieh

**Affiliations:** 1 Department of Chinese Pharmaceutical Sciences and Chinese Medicine Resources, China Medical University, Taichung 404333, Taiwan; shangchyi@gmail.com (M.-H.H.); yschang@mail.cmu.edu.tw (Y.-S.C.); 2 School of Pharmacy, China Medical University, Taichung 406040, Taiwan; mildds@yahoo.com.tw; 3 Institute of Bioinformatics and Structural Biology, National Tsing-Hua University, Hsinchu 300044, Taiwan; pclyu@mx.nthu.edu.tw; 4 Institute of Population Sciences, National Health Research Institutes, Hsinchu 35053, Taiwan; jong212@gmail.com; 5 Department of Biological Science and Technology, China Medical University, Taichung 406040, Taiwan; jgchung@mail.cmu.edu.tw; 6 Graduate Institute of Integrated Medicine, College of Chinese Medicine, China Medical University, Taichung 40402, Taiwan; linwy@mail.cmu.edu.tw; 7 Department of Pharmacology, China Medical University, Taichung 404333, Taiwan; 8 Chinese Medicine Research Center, China Medical University, Taichung 404333, Taiwan

**Keywords:** imperatorin, *Notopterygium incisum*, anti-inflammatory effects, LPS/TLR4 signal transduction, Nrf2 antioxidative pathway, protein-ligand docking assay

## Abstract

Imperatorin (IMP) could downregulate several inflammatory transcription factor signaling pathways. Some studies have pointed out that IMP could interfere with toll-like receptor 4 (TLR4) signaling. This study evaluates how IMP interferes with the TLR4 co-receptors signaling through the protein-ligand docking model, Western blotting, immunofluorescence (IF), and atomic force microscopy (AFM) assays in lipopolysaccharide (LPS) stimulated macrophage-like RAW264.7 cells in vitro. The results of the protein-ligand docking demonstrate that IMP interferes with LPS binding to the LPS-binding protein (LBP), the cluster of differentiation 14 (CD14), and the toll-like receptor 4/myeloid differentiation factor 2 (TLR4/MD-2) co-receptors in LPS-stimulated RAW264.7 cells. Compared with TLR4 antagonist CLI-095 or dexamethasone, IMP could suppress the protein expressions of LBP, CD14, and TLR4/MD-2 in LPS-stimulated cells. Furthermore, the three-dimensional (3D) image assay of the AFM showed IMP could prevent the LPS-induced morphological change in RAW264.7 cells. Additionally, IMP could activate the nuclear factor erythroid 2-related factor 2 (Nrf2) signaling pathway, and it increased the antioxidative protein expression of heme oxygenase-1 (HO-1), superoxidase dismutase (SOD), and catalase (CAT). Our results are the first to reveal that the anti-inflammatory effect of IMP interferes with LPS binding to TLR4 co-receptor signaling and activates the antioxidative Nrf2 signaling pathway.

## 1. Introduction

Previous reports’ data suggest that Toll-like receptor 4 (TLR4) could be the targeted therapeutics for immunopharmacological regulators of infectious and inflammatory diseases [1]. However, lipopolysaccharide (LPS) is the primary inflammatory pathogen. LPS is recognized in conjunction with the LPS-binding protein (LBP) and transfers to the differentiation 14 (CD14) co-receptor. Then, LPS translocates to the TLR4/myeloid differentiation factor 2 (MD-2) complexing with the transmembrane domain co-receptor and initiates the inflammatory signaling [2,3]. TLR4 activates transcription factors in the intracellular space, such as nuclear factor-κB (NF-κB), activator protein 1 (AP-1), and signal transducer and activator of transcription 3 (STAT3), regulates various inflammatory genes, and activates many infectious and noninfectious diseases [4,5,6]. Several studies have noted that TLR4 expression of isolated monocytes in the presence of inflammatory cytokines such as tumor necrosis factor-α (TNF-α), interleukin 2 (IL-2), interleukin 6 (IL-6), interleukin 8 (IL-8), and interleukin 10 (IL-10) triggers the JAK/STAT signaling pathways [7]. Moreover, many approaches have developed TLR4 antagonists such as TAK-242 (CLI-095), which have advanced to block TLR4 signaling in various diseases such as sepsis, septic shock, rheumatoid arthritis, and lung inflammation [8]. Additionally, dexamethasone (Dexa) treatment could reduce the expression levels of TLR4 and MyD88 [9]. Therefore, the modulation of TLR4 activity is a novel target for the TLR4-mediated inflammatory response, immune disease, metabolic disease, and cancer [10].

It is crucial to notice that TLR4 signal crosstalk with the Nuclear factor erythroid 2-related factor (Nrf2) pathway plays a role in the restoration of internal anti-inflammatory defense and tissue balance after inflammation occurs [11,12]. Previous studies have provided evidence that Nrf2/Heme oxygenase-1 (HO-1) is an antioxidant pathway and that the transcription factor Nrf2 suppresses oxidative stress and induces anti-inflammatory effects in macrophages [13]. Specifically, the phosphatidylinositol 3-kinases (PI3K)/Akt pathway regulates the Nrf2-dependent defense against oxidative stress [14]. In addition, Nrf2 is a transcription factor that is related to the induction of cytoprotective proteins of HO-1, glutathione peroxidase (GPx), superoxidase dismutase (SOD), and catalase (CAT), allowing the elimination of free radicals in cells caused by oxidative damage [15]. Another notable finding is that higher amounts of Nrf2 increased translocation into the nucleus and decreased the cytoplasm protein expression, increasing the antioxidative molecules’ expression [16].

In this study, imperatorin (IMP) was isolated from *Notopterygium incisum.* However, IMP is distributed in many natural plants and produces anti-inflammatory activity [17]. IMP has been reported as having anti-inflammatory activity. It could downregulate several signaling pathways, including PI3K/Akt, extracellular regulated protein kinase (ERK), reactive oxygen species (ROS), and TNF-α [18,19]. Moreover, IMP suppresses the IκB kinase (IKK)/nuclear factor-κB pathways [20,21]. In addition, IMP can inhibit pro-inflammatory mediators, including inducible nitric oxide synthase (iNOS) and cyclooxygenase 2 (COX-2). IMP can reduce pro-inflammatory mediators and cytokines via inhibited NF-κB and JAK/STAT signaling pathways in vitro [22]. However, IMP could regulate the Keap-1/Nrf2/HO-1-mediated signaling pathways [23,24]. Moreover, IMP stimulates antioxidant production, including CAT, SOD, and GXs [25]. In computer docking modeling, IMP binds to Keap-1, forms a hydrogen bond, interacts with NH2 of the side chain of N414, and forms two hydrogen bonds with the side chain of S602 and S363 [26]. Moreover, IMP exhibits low bioavailability and has poor intracellular absorption [27]. Therefore, IMP may also occur via the surface receptor to activate downstream signaling pathways.

The fact that IMP can influence serval transcription factors shows that its influence is related to the upstream TLR4 signaling pathway. Therefore, this study aimed to investigate anti-inflammatory effects affecting the TLR4 signaling of IMP in LPS-stimulated RAW264.7 murine macrophage cells in vitro.

## 2. Materials and Methods

### 2.1. Materials and Reagents

IMP was isolated from *Notopterygium incisum*. Lipopolysaccharides (LPS), Dimethyl sulfoxide (DMSO), Dexamethasone (Dexa), Sodium nitrite, Paraformaldehyde, Bovine serum albumin (BSA), sulfanilamide, Phosphoric acid, N-1-naphthyl ethylenediamine dihydrochloride, Sodium dodecyl sulfate-polyacrylamide gel (SDS-PAGE), 3-(4,5-Dimethylthiazol-2-yl)-2,5-diphenyltetrazolium bromide reagent (MTT), *N*-1-naphthyl ethylenediamine dihydrochloride, Sulfanilamide, Phosphoric acid, Fetal bovine serum (FBS), Penicillin, and streptomycin were purchased from Sigma-Aldrich (St. Louis, MO, USA). Dulbecco’s modified Eagle’s medium (DMEM) was purchased from Gibco (Grand Island, NY, USA). ELISA Kits of IL-1β, IL-6, TNF-α, TRIzol™ Reagent, SuperScript™ II Reverse Transcriptase, RNaseOUT™ Recombinant RNase Inhibitor, Hoechst 33258, Alexa Fluor 488, and Alexa Fluor 594 were purchased from Invitrogen (Carlsbad, CA, USA). Primary antibody anti-Akt, p-Akt, Nrf2, SOD1, SOD2, CAT, and HO-1 were purchased from Cell Signaling (Beverly, MA, USA). PGE_2_ ELISA kit was purchased from Cayman (Ann Arbor, MI, USA). Enhanced chemiluminescence (ECL) was purchased from Perkin Elmer (Waltham, MA, USA). IRdye-labeled NF-κB oligonucleotide was purchased from LI-COR Biosciences (Lincoln, NE, USA).

### 2.2. Protein–Ligand Docking Analysis

The protein–ligand molecular modeling and computational studies provided insights into the TLR4 co-receptor mechanism and the essential interactions modulating the molecular recognition process of agonist and antagonist ligands [28]. Moreover, recent works indicated that Dexa docked into the DNA binding region of the NF-κB p50 with hydrogen bonding interactions [29]. Virtual molecular screening is usually used for docking small molecules, using the PyRx/AutoDock Vina assembled on the Lamarckian genetic algorithm and the empirical free energy score function [30]. The receptor structures, including the LPS-binding protein (LBP) (PDB: 4M4D), Cluster of differentiation 14 (CD14) (PDB: 4GLP), and TLR4/MD-2 (Myeloid differentiation factor 2) complex (PDB: 3FXI), were obtained from the RCSB Protein Databank [3]. The ligand structures of IMP and Dexa were obtained from PubChem CID: 10,212 and CID: 28,932. It was necessary to set the docking search space of LBP at the N-terminal domain, the primary interaction with LPS [31]. Moreover, we set the docking search space of CD14 at the N-terminal hydrophobic pocket side, which functions with the binding and delivery of various lipid molecules, including LPS [32]. Furthermore, we set the docking search space of the TLR4/MD-2 complex at the LPS binding site of the 3FXI structure [33]. In comparison, the TLR4 antagonists docked in the hMD-2 simulation modeling show that TLR4 can interact with Tyr102 and Arg90 of MD-2 and obtain a stable complex that interferes with LPS in the same binding site of MD-2 [28]. Consequently, docked complexes were visualized and analyzed using the PyMOL Molecular Graphics System (Ver. 2.3 Schrödinger, Portland, OR, USA), and the interactions between protein and ligand were analyzed using the "LIGPLOT" (a program to generate schematic diagrams of protein-ligand interactions) module within the LigPlot+ program (v2.2) [34]. Another possible explanation for the result is that the docking assay was used to analyze the protein-ligand complex binding affinity (kcal/mol). The affinity value changed more negatively and provided compelling evidence of ligand interaction in the binding site [35].

### 2.3. Cell Culture

RAW264.7 murine macrophage cells were obtained from the Food Industry Research and Development Institute (Hsinchu, Taiwan). RAW264.7 cells were cultured in Dulbecco’s modified Eagle’s medium supplemented with 10% FBS, 100 U/mL of penicillin, and 100 μg/mL streptomycin. Cells were grown at 37 °C in incubators in a humidified 5% CO_2_ condition.

### 2.4. Atom Force Microscopy Analysis

AFM is an innovative tool for measuring molecular–molecular interaction forces and capturing high-resolution images [36]. Cells were incubated in glass slides and treated with IMP or Dexa and then cells were fixed with 4% paraformaldehyde. We determined the desired force profile (amplitude and force load rate) with AFM, with the cantilever deflection, dc (t), and the cantilever fixed end, zc (t). The probe was an APP-Nano ACTA series where the radius of the tip and the cantilever spring constant were below 7 nm and 7 N/m. We set the desired force profile at a 1 Hz triangle wave of amplitude 0.2–0.3 nN, with a duration of 600–1200 s (to monitor the time-elapsed evolution of Young’s modulus). Moreover, in some scanning parameters, the tip scan rate was 0.5–1 Hz, and the resolution of images was 512 by 512 pixels. From the AFM image, the superstructure is significant, surrounding the nucleus of macrophages. The binding force was measured with the Bruker Dimension Icon AFM (Bruker Corporation, Billerica, MA, USA). The cell ultrastructure morphology was analyzed with the NanoScope analysis software version 7 of the instrument (Bruker Dimension Icon, Santa Barbara, CA, USA).

### 2.5. Immunofluorescence Assay

An immunofluorescence assay allows visualization of the distribution of target molecules through the fluorescence microscope, as described previously [37].

RAW 264.7 cells were incubated in a confocal laser slide in the dish at 500 cells/well for 16 h and treated with IMP or Dexa for various periods before being incubated with 100 ng/mL LPS. Then, cells were fixed in 4% paraformaldehyde and permeabilized with 0.25% Triton X-100 in PBS for 1 h. Moreover, cells were incubated with the primary antibodies overnight at 4 °C and then complemented with a secondary antibody labeled with an IgG Alexa Fluor-594 reagent. Then, we stained the nuclei with 4′, 6-diamidino-2-phenylindole (DAPI) gel (1 μg/mL). The immunofluorescence assay was visualized with an SP2/SP8X Confocal Spectral Microscope (Leica, Wetzlar, Germany).

### 2.6. Western Blot assay

Western blot is a critical technique used to identify specific proteins from a complex mixture of proteins in the cells described previously [38]. Cells were treated with IMP and LPS and lysed with RIPA buffer to extract the protein from the cells in each group. The bicinchoninic acid (BCA) assay was used to detect the protein concentration. The proteins were then separated with 8–12% SDS-PAGE electrophoresis and transferred to PVDF membranes. The membranes were probed with primary antibodies and incubated with horseradish peroxidase (HRP)-conjugated secondary antibody. Immune complexes were visualized with an enhanced chemiluminescence reagent. We then quantified the proteins with the GE Las4000 Mimi Molecular Imaging System (GE healthcare co, Piscataway, NJ, USA) and explored the data from each group using the TotalLab gel analysis software (GE healthcare co, Piscataway, NJ, USA).

### 2.7. Statistical Analysis

All experimental data are presented as the mean ± standard error of the mean (SEM). An unpaired Student’s *t*-test or the SPSS17.0 software system was used for a one-way analysis of variance to determine the statistical significance. Differences between the LPS-treated and control groups were considered statistically significant at the level of ^#^ *p* < 0.05 compared with the control group, * *p* < 0.05, ** *p* < 0.01, and *** *p* < 0.001 compared with the LPS-alone group.

## 3. Results

### 3.1. IMP Interfered with the LBP, CD14, and MD-2 in TLR4 Co-Receptor Complex with the Computational Protein-Ligand Docking Model

**Figure 1:**
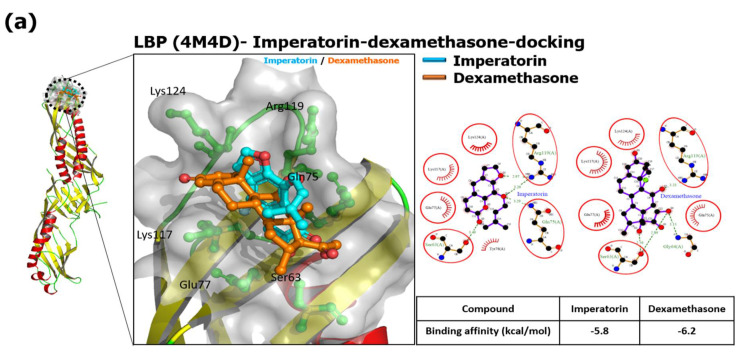

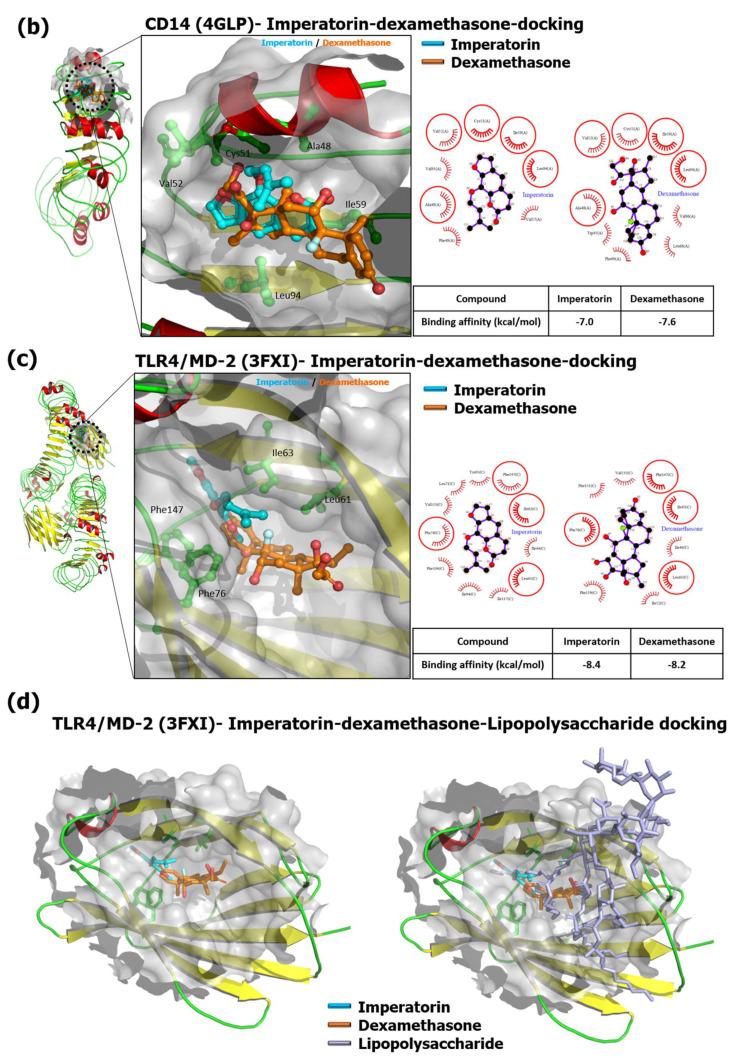
IMP interfered with the LBP, CD14, and MD-2, in TLR4 co-receptor complex with the computational protein–ligand docking model. The binding pocket side is a surface model, and the inhibitor is a ball and stick model. Red or pink eyebrow-like icons illustrate hydrophobic interactions. The green dash line indicates the hydrogen bonds pairing with each other. The red circles identify the residue on each plot that is equivalent. The best binding affinities between IMP and Dexa to LBP are shown below. LPS is a gray color. IMP is a light blue color. Dexa is an orange color. (**a**) LBP binding with IMP and Dexa (superimposition). The LigPlot+ 2D diagrams show the potential intermolecular interactions in IMP/LBP and Dexa/LBP. (**b**) CD14 binding with IMP and Dexa (superimposition). The LigPlot+ 2D diagrams of the potential intermolecular interactions in IMP/CD14 and Dexa/CD14. The best binding affinities of IMP and Dexa were in the binding of CD14. (**c**) TLR4/MD-2 binding with IMP and Dexa (superimposition). The LigPlot+ 2D diagrams of the potential intermolecular interactions in IMP/MD-2 and Dexa/MD2. (**d**) Structures of IMP or Dexa bound to MD-2 influence the LPS binding to the TLR4/MD-2 co-receptor complex.

In the present study, the results show that IMP and Dexa bind the N-terminal pocket side of LBP. Both are surrounded by Ser63, Gln75, Glu77, Lys117, Arg119, and Lys124, found in the LIGPLOT analysis with a hydrophobic interaction. We prepared the receptor structure and ligands, and then docking was performed into a grid box space with an X-, Y-, and Z-axis, and dimensions were adjusted to 41.53 Å × 38.36 Å × 45.21 Å, 48.53 Å × 36.41 Å × 45.26 Å, and 43.25 Å × 31.49 Å × 35.17 Å within LBP, CD14, and TLR4/MD-2 complex, respectively. A data set was generated from the docking search space of LBP which was at the N-terminal domain. LBP has a highly extended structure that is 33 Å wide and 127 Å long. However, the binding affinities of IMP and Dexa on the N-terminal pocket side of LBP are −5.8 and −6.2 kcal/mol, respectively (Figure 1a). The signals collected from IMP or Dexa mainly bind to the pocket side of CD14 through hydrophobic interaction, and the common residues are Ala48, Cys51, Val52, Ile59, and Leu94 on the N-terminal pocket side. Furthermore, IMP has other hydrophobic interactions (residues Phe49, Val57, Val91) when binding to CD14. Dexa also has other hydrophobic interactions (residues Trp45, Leu66, Phe69, and Val96) when binding to CD14. Regarding PyRx docking, the binding affinities of IMP and Dexa on the N-terminal hydrophobic pocket side of CD14 are −7.0 and −7.6 kcal/mol, respectively (Figure 1b). The data acquired from the docking results show that IMP or Dexa binds deep in the MD-2 pocket side. In addition to the common residues (Leu61, Ile63, Phe76, Phe147) of the hydrophobic interaction, IMP also has a hydrophobic interaction with residues Ile44, Tyr65, Leu71, Ile94, Phe104, Val113, and Ile117. A data set generated from Dexa also showed a hydrophobic interaction with Ile46, Ile52, Phe119, Val135, and Phe151. Considering PyRx docking, the binding affinities of IMP and Dexa on the N-terminal hydrophobic pocket side of MD-2 are −8.4 and −8.2 kcal/mol, respectively (Figure 1c). These results show that the binding of IMP or Dexa will occupy space and affect the binding of LPS in the MD-2 pocket side when comparing the docked complex and LPS binding site of the 3FXI structure (Figure 1d).

### 3.2. IMP Interferes with LPS Binding to TLR4 Co-Receptor Complex in RAW264.7 Cells

**Figure 2:**
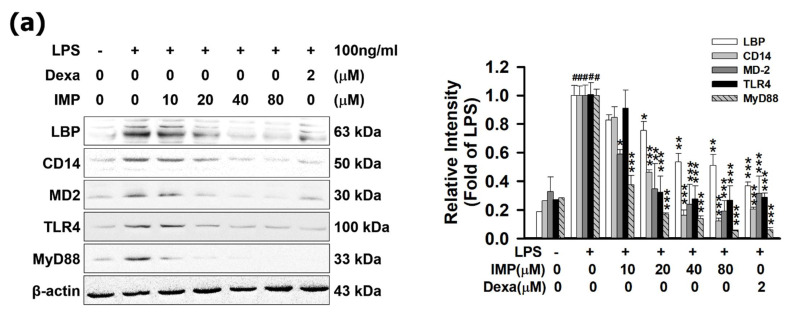

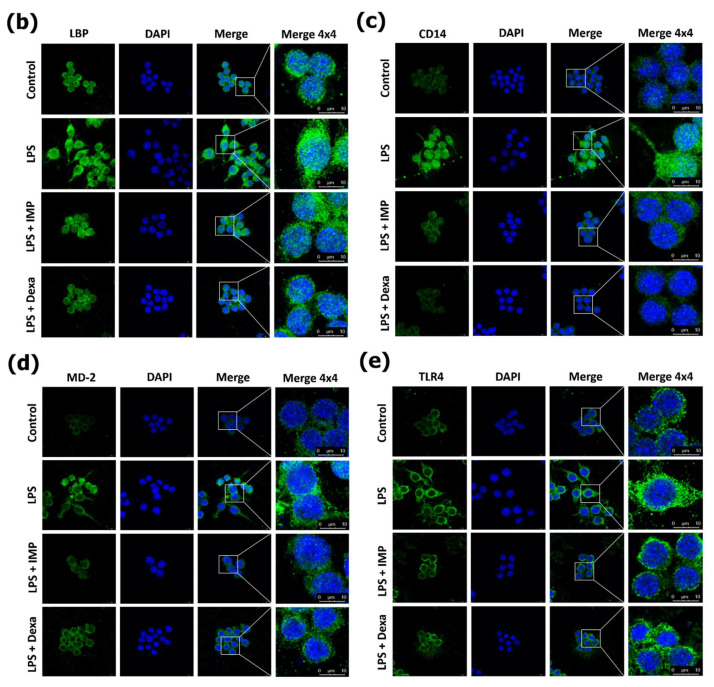
IMP interferes with LPS binding to LBP, CD14, and MD-2, in TLR4 co-receptor complex in RAW264.7 cells. RAW264.7 cells were pretreated with IMP for 1 h and then stimulated without or with 100 ng/mL of LPS for 24 h incubation. (**a**) Cells were pretreated with various IMP concentrations (0, 10, 20, 40, or 80 μM) or Dexa (2 μM) for 1 h and were stimulated with LPS for 24 h. The protein expressions of LBP, CD14, MD-2, TLR4, and MyD88 were measured by Western blotting (*n* = 3). (**b**) Cells were pretreated with 80 μM IMP or 2 μM Dexa for 1 h and were stimulated with LPS for 24 h. We also measured LBP by immunofluorescence staining. For LBP (green) in cells, nuclei were labeled with DAPI (blue). Alternatively, it was stained for CD14 (green) (**c**), or cells were stained for MD-2 (green) (**d**) and TLR4 (green) (**e**). Scale bar = 10 μm. The detailed experimental preforms are described in Materials and Methods (*n* = 3). Results (data) are presented as means ± S.E.M. ^#^ *p* < 0.05 compared with the control group, * *p* < 0.05, ** *p* < 0.01, and *** *p* < 0.001 compared with the LPS-alone group.

In the experiment shown in Figure 2a, IMP suppressed LBP, CD14, MD-2, and TLR4 expressions in a dose-dependent manner. Comparing Dexa (2 μM) and IMP (80 μM), it is shown that IMP inhibited the protein expression ratios of LBP, CD14, MD-2, and TLR4, which were 63.21% (0.63-fold), 79.41% (0.79-fold), 68.50% (0.68-fold), and 71.10% (0.71-fold), respectively. Moreover, this experiment shows that IMP could decrease LBP, CD14, MD-2, and TLR4 in the cellular membrane surface with immunofluorescence staining (Figure 2b–e). One possible explanation for this result is that IMP has anti-inflammatory activity by modulating the TLR4/MyD88 cascade signaling pathways.

### 3.3. IMP Inhibited the Morphological Change in LPS-Stimulated RAW264.7 Cells

**Figure 3:**
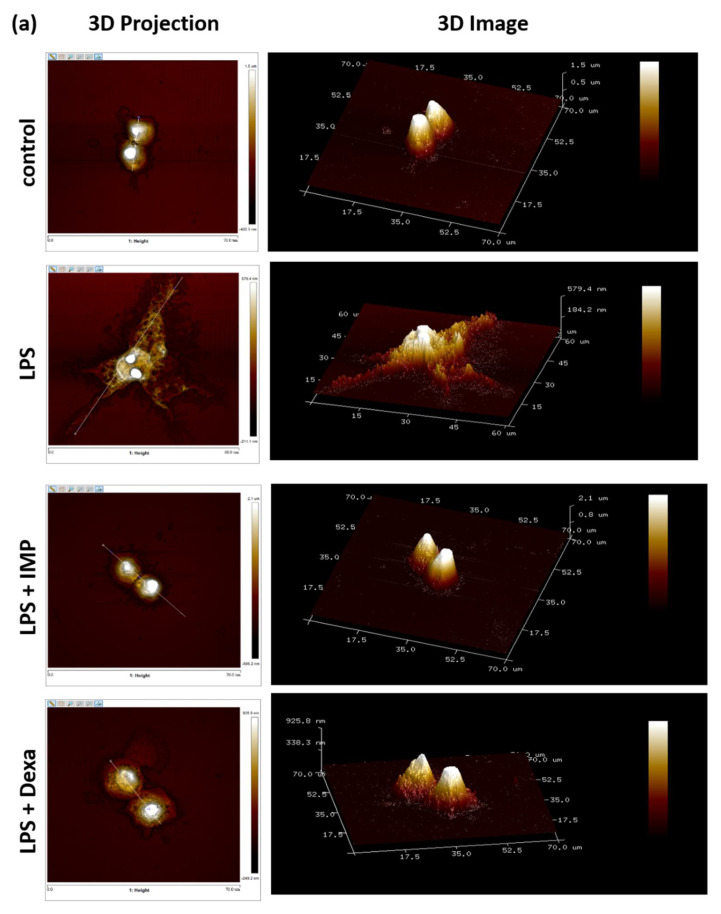

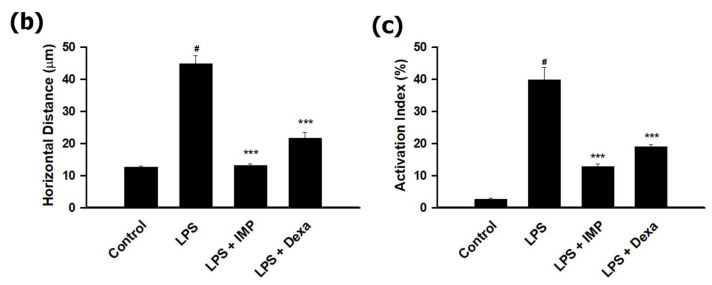
IMP inhibited the morphological change in LPS-stimulated RAW264.7 cells. Cells were cultured at a density of 5 × 10^4^ cells/mL on glass coverslips in a six-well plate. After overnight incubation, IMP was incubated without or with 100 ng/mL LPS into the cell culture medium for 24 h of incubation. (**a**) The surface ultrastructural change assay by AFM. AFM was applied to observe the surface ultrastructure changes in the present work, including amplitude error, 3D projection from upside, and 3D images. The LPS-alone group was the group where cells were only treated with LPS. The dendritic transformation assay and surface ultrastructural change assay were detected by atomic force microscopy, as designated in the Materials and Methods (*n* = 4). Topography presented as 3D height sensor and the horizontal distance of RAW264.7 cells. (**b**) LPS increases the activation index of the morphological change in cells. (**c**) The horizontal distance of cell ultrastructure with the instrument’s NanoScope analysis software. Data are accessible as the means ± S.E.M. ^#^ *p* < 0.05 as compared with the control group; *** *p* < 0.001 compared with the LPS-alone group.

The experiments were designed to provide information from the AFM assay, and which it can deliver the high-resolution 3D imaging information of morphological and precise membrane features, as well as ultrastructural changes in cells, which can be detected at the nanoscale. The test sequence was wholly randomized and counterbalanced to reduce error. To ensure repeatable and stable characteristics, we calculated the top four morphological changes in cells in slides of each group. Cells were incubated in glass slides and treated with IMP or compared with the 2 μM of Dexa and treated with 100 ng/mL of LPS for 24 h of incubation. As shown in Figure 3a, the cells revealed a typical oval shape with a smooth cell surface and lamellipodia formation. The horizontal distance is about 13.6 ± 0.5 μm in the control group (without added LPS). However, LPS increased the horizontal distance near 59.1 ± 3.3 μm in the RAW264.7 cells (*n* = 4). This experiment showed that after pretreatment with IMP (80 μM), it showed the typical oval shape with the smooth cell surface, and the horizontal distance was 13.6 ± 0.4 μm (Figure 3a). In LPS-stimulated RAW264.7 cells, pretreatment with 2 μM Dexa reversed the pseudopodia formation. The horizontal distance in the Dexa group was approximately 19.4 ± 1.97 μm (Figure 3a). The most notable changes were associated, after exposure, with 24 h of 100 ng/mL of LPS, and the activation index of the LPS-only group, IMP group, and Dexa group is 38.70%, 10.60%, and 18.92%, respectively (Figure 3b). Regarding these results, the horizontal distances of the LPS-only group, the IMP group, and the Dexa group are 44.80%, 13.07%, and 21.67%, respectively (Figure 3c). Comparing the data from the LPS-only group and the Dexa group shows that IMP inhibited lamellipodia formation and reversed the horizontal distance in the LPS-stimulated RAW264.7 cells.

### 3.4. IMP Activated the Nrf2/HO-1 Pathways in LPS-Stimulated RAW264.7 Cells

**Figure 4:**
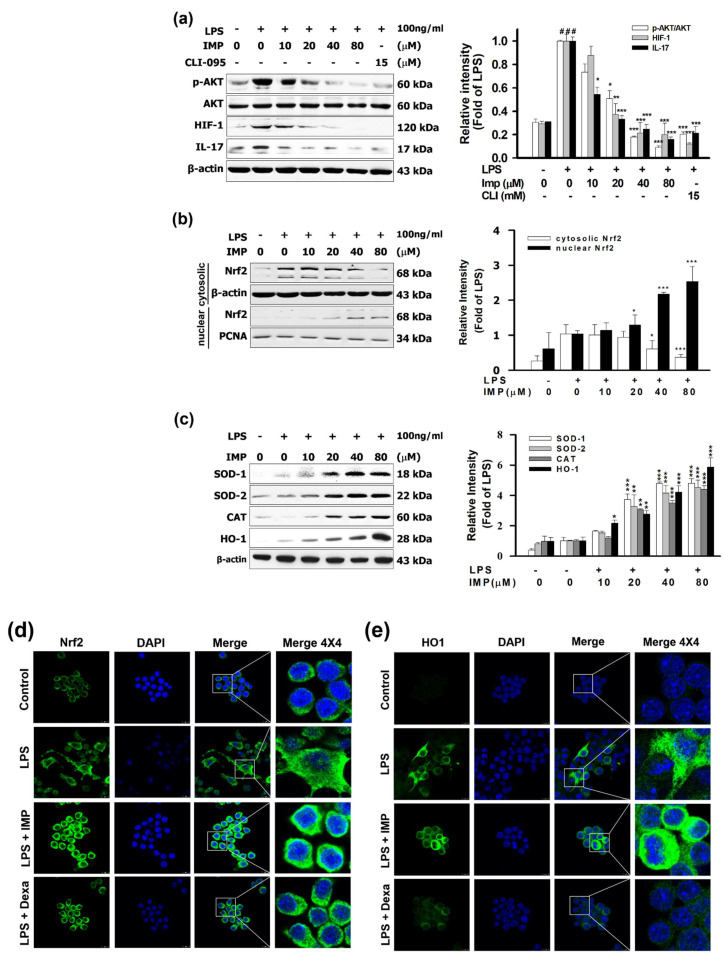
IMP activated the Nrf2/HO-1 pathways in LPS-stimulated RAW264.7 cells. RAW264.7 cells were pretreated with drugs and then stimulated with 100 ng/mL of LPS for 24 h of incubation. (**a**) Cells were pretreated with various IMP concentrations (0, 10, 20, 40, or 80 μM) or 15 μM of CLI-095 for 1 h and then stimulated without or with LPS. The p-AKT, HIF-1, and IL-17 were measured by Western blotting in the cytoplasm and nucleus (*n* = 3). (**b**) Cells were pretreated with various IMP concentrations (0, 10, 20, 40, or 80 μM) for 1 h and then stimulated without or with LPS. Nrf2 was measured by Western blotting in the cytoplasm and nucleus (*n* = 3). (**c**) Cells were pretreated with various IMP concentrations (0, 10, 20, 40, or 80 μM) for 1 h and then stimulated without or with LPS. SOD1, SOD2, CAT, and HO-1 were measured by Western blotting (*n* = 3). (**d**) Cells were pretreated with IMP (80 μM) and then stimulated without or with 100 ng/mL of LPS. Nrf2 was measured by immunofluorescence staining. For Nrf2 (green) in RAW264.7 cells treated with LPS for 24 h, nuclei were labeled with DAPI (blue). (**e**) Cells were pretreated with IMP (80 μM) and then stimulated without or with 100 ng/mL of LPS. HO-1 was measured by immunofluorescence staining. For HO-1 (green) in cells treated with LPS for 24 h, nuclei were labeled with DAPI (blue). All the methods are designated in the Materials and Methods (*n* = 3). Data are accessible as the means ± S.E.M. ^#^ *p* < 0.05 compared with the control group, * *p* < 0.05, ** *p* < 0.01, and *** *p* < 0.001 compared with the LPS-alone group.

It is reasonable that, compared with the 15 μM of CLI-095, the TLR4 antagonist, IMP (80 μM) inhibited the ratio of phosphorylation of AKT at 90.99%, inhibited the HIF-1 ratio at 65.18%, and inhibited the IL-17 ratio at 80.38%. However, as shown in Figure 4a, IMP significantly stimulated the LPS-induced cytosolic Nrf2 protein expression. In contrast, the results demonstrate that IMP (80 μM) increased the nuclear translocation factor of Nrf2 in the cytoplasm to 88.84%, but Nrf2 protein expression in the nucleus increased more than 293.79% (2.93-fold) (Figure 4b). Moreover, IMP (80 μM) increased the antioxidant expression of SOD1, SOD2 CAT, and HO-1 to 480.62%, 451.02% (4.51-fold), 440.86% (4.40-fold), and 586.89% (5.86-fold), respectively (Figure 4c). The data from the immunofluorescence assay show that IMP decreased the Nrf2 protein expression in the cytoplasm but increased the translocation and the accumulation of Nrf2 into the nucleus (Figure 4d). These results are consistent with the immunofluorescence assay results that showed that IMP could increase HO-1 in the cytoplasm and increase translocation and accumulation of HO-1 in the cytoplasm (Figure 4e). These results suggest that IMP increased the expression and translocation of the antioxidation transcription factor Nrf2 and activated the antioxidant process of SOD1, SOD2 CAT, and HO-1.

## 4. Discussion

Imperatorin (IMP) has been broadly used in various applications, including anti-cancer, neuroprotection, anti-inflammatory, anti-hypertension, and antibacterial studies [27]. IMP has been reported to act with anti-inflammatory activity via downregulating several inflammatory transcription factors [18,19]. However, IMP exhibits low bioavailability and has poor intracellular absorption. Therefore, IMP may also act via the surface receptor to activate downstream signaling pathways. More evidence has emerged that IMP could interfere with TLR4 signaling.

Cell staining, flow cytometry, cell surface biotinylation, immunoprecipitation, and immunoprobing assays are usually used to detect the interaction between the ligand and the binding protein [39]. We simulated the binding interactions between proteins (LBP, CD14, TLR4/MD-2 complex) and ligands (IMP and Dexa) by the protein–ligand docking software PyRx, as previously described [40]. Moreover, the N-terminal domain is the primary site of interaction with LPS, particularly the positively charged residues at its tip, including residues Arg119, Lys120, and Lys124 [31]. The data were acquired from the pocket side containing the N-terminal region (residues 20-171) of CD14, sufficient for bioactivity, which serves as the binding site for LPS [32]. In the present study, in the IMP–LBP docking analysis result, IMP significantly interacted with Arg119 and Lys124 of the LBP relative position, and Arg119 generated hydrogen bonds with IMP that could interfere with the LPS binding to the LBP. Moreover, this experiment’s results show IMP docked in the hydrophobic pocket side of CD14, around residues Ala48, Cys51, Val52, Ile59, and Leu94. The binding of IMP to CD14 may hinder the transfer of LPS to another molecule, such as MD-2 (Figure 1b). The most notable changes were associated with IMP docked deep inside the pocket side of the MD-2 structure that affects the binding of LPS to TLR4/MD-2, since lipid chains of LPS cannot extend to the MD-2 hydrophobic pocket side to form stable hydrophobic interactions. Additionally, the binding affinity of IMP is lower than that of the Dexa in LBP, CD14, and TLR4/MD-2. IMP or Dexa bound to MD-2 influences the LPS bound to the TLR4/MD-2 co-receptor complex. The simulation suggests that IMP could affect LPS by disturbing the LBP binding, transporting to CD14, and lodging MD-2 in the TLR4 transmembrane domain co-receptor complex. The results indicate that IMP could decrease LBP, CD14, MD-2, and TLR4 in the cellular membrane surface with immunofluorescence staining.

Our primary objectives in the AFM assay were to evaluate the LPS-stimulated distinct dendritic morphology change and increased cell size in RAW2647 cells [41]. According to the report, LPS significantly increases the activation index and the morphological change in cells [42]. The most notable changes were associated with IMP suppressing LBP, CD14, MD-2, and TLR4 expressions in a dose-dependent manner. Moreover, the Results data also indicate that IMP could decrease LBP, CD14, MD-2, and TLR4 in the cellular membrane surface with immunofluorescence staining. It could be established that IMP has anti-inflammatory activity by modulating the TLR4/MyD88 cascade signaling pathways. The performance trends in evaluating the proposed LPS-stimulated distinct dendritic morphology change and increased cell size in RAW2647 cells can be detected at the nanoscale in the AFM assay. The horizontal distance of LPS increased by nearly 4.3-fold (59.1 ± 3.3 μm) in the RAW264.7 cells in the AFM assay. However, we can notice that IMP showed a reversal of the typical oval shape with the smooth cell surface and the horizontal distance of LPS, compared with LPS alone, or Dexa in LPS stimulated. These results show that it is similar to what was achieved in the activation index changes. Regarding these similar results, in the horizontal distance analysis, comparing with Dexa shows that IMP inhibited lamellipodia formation and reversed the horizontal distance in LPS-stimulated RAW264.7 cells. The morphological analysis observations indicated that IMP could inhibit lamellipodia formation by reversing the LPS-stimulated RAW264.7 cells’ size.

Moreover, LPS/TLR4 signaling triggered Akt phosphorylation, increased hypoxia-inducible factor 1 (HIF-1) expression, and activated interleukin 17 (IL-17)-induced tissue inflammation [43,44]. However, the PI3K/Akt signaling pathway can regulate the expression of hypoxia-induced factor-1α (HIF-1α) [45]. HIF-1 improves TH17 advancement through direct transcriptional activation in a STAT3-dependent manner [46,47]. It is reasonable that compared with the 15 μM of CLI-095, the TLR4 antagonist, IMP inhibited the ratio of phosphorylation of AKT and inhibited HIF-1 and IL-17 protein expressions. PI3K/Akt pathway signaling moderates the NRF2/Keap-1 signaling to protect the cells and tissues from oxidative stress [48,49]. Several phytochemical compounds exhibit anti-inflammatory effects and up-regulate the AMPK/GSK-3β/Nrf2 pathway [16,50,51]. Nrf2 interferes with LPS-induced transcription, increasing the pro-inflammatory cytokines. Immunoprecipitation assays showed that Nrf2 binds to the nearness of these genes in macrophages [13]. As shown in Figure 4a, IMP significantly stimulated the LPS-induced cytosolic Nrf2 protein expression. Moreover, IMP dramatically increases the antioxidant process of SOD1, SOD2 CAT, and HO-1. The data confirmed that IMP could increase SOD from the IF assay. These results are consistent with the IF assay results showing that IMP could increase HO-1 in the cytoplasm and increase translocation and accumulation of HO-1 in the nucleus. These results suggest that IMP increased the expression and translocation of the antioxidation transcription factor Nrf2 and activated the antioxidant process of SOD1, SOD2 CAT, and HO-1.

## 5. Conclusions

**Figure 5:**
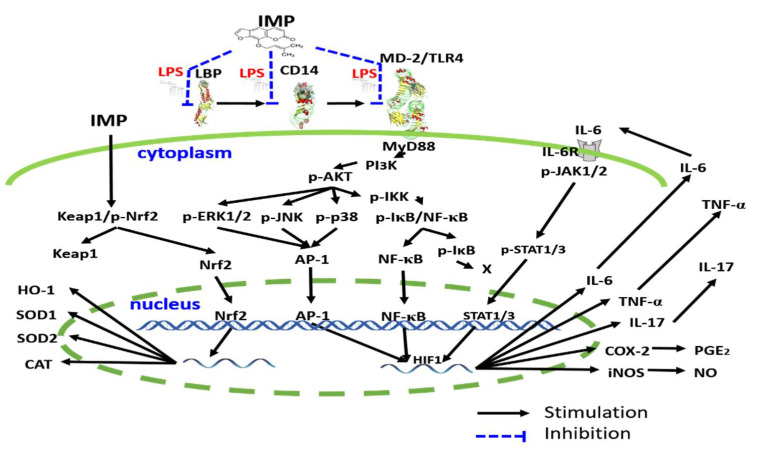
The proposed mechanism is depicting the effect of IMP inhibiting inflammatory effects. IMP could interfere with the LPS binding to LBP, CD14, and MD-2 on the TLR4 co-receptor complex. Moreover, according to the previous reports described and the results in this manuscript, IMP attenuates the inflammatory effect cascade via inhibition of transcription factors including IκB/NF-κB, MAPK/AP-1, and JAKs/STATs inflammatory transcription factor signaling pathways. Moreover, IMP activates the antioxidative transcription factor Nrf2 signaling pathway in LPS-induced RAW264.7 murine macrophage cells.

Our results are the first findings regarding the anti-inflammatory effect of IMP that interfered with the LPS binding to the TLR4 co-receptor signaling. They also show that IMP activated the antioxidative Nrf2 signaling pathway in LPS-induced RAW264.7 murine macrophage cells in vitro. Overall, the possible molecular signaling pathways of IMP in our results are summarized in Figure 5.

## Data Availability

Data is contained within the article.
